# Temporal dynamics and transcriptional control using single-cell gene expression analysis

**DOI:** 10.1186/gb-2013-14-10-r118

**Published:** 2013-10-24

**Authors:** Tsukasa Kouno, Michiel de Hoon, Jessica C Mar, Yasuhiro Tomaru, Mitsuoki Kawano, Piero Carninci, Harukazu Suzuki, Yoshihide Hayashizaki, Jay W Shin

**Affiliations:** 1 RIKEN Center for Life Science Technologies, Division of Genomic Technologies, 1-7-22 Suehiro-cho, Tsurumi-Ku, Yokohama 230-0045, Japan; 2 RIKEN Omics Science Center, Yokohama 230-0045, Japan; 3 Department of Systems & Computational Biology, Albert Einstein College of Medicine, Bronx, NY 10461, USA; 4 RIKEN Preventive Medicine and Diagnosis Innovation Program, Wako, Saitama 351-0198, Japan

## Abstract

**Background:**

Changes in environmental conditions lead to expression variation that manifest at the level of gene regulatory networks. Despite a strong understanding of the role noise plays in synthetic biological systems, it remains unclear how propagation of expression heterogeneity in an endogenous regulatory network is distributed and utilized by cells transitioning through a key developmental event.

**Results:**

Here we investigate the temporal dynamics of a single-cell transcriptional network of 45 transcription factors in THP-1 human myeloid monocytic leukemia cells undergoing differentiation to macrophages. We systematically measure temporal regulation of expression and variation by profiling 120 single cells at eight distinct time points, and infer highly controlled regulatory modules through which signaling operates with stochastic effects. This reveals dynamic and specific rewiring as a cellular strategy for differentiation. The integration of both positive and negative co-expression networks further identifies the proto-oncogene *MYB* as a network hinge to modulate both the pro- and anti-differentiation pathways.

**Conclusions:**

Compared to averaged cell populations, temporal single-cell expression profiling provides a much more powerful technique to probe for mechanistic insights underlying cellular differentiation. We believe that our approach will form the basis of novel strategies to study the regulation of transcription at a single-cell level.

## Background

Genetically identical cells exposed to the same environmental factors can elicit significant variation in gene expression and phenotype [1]. This variability is sourced to stochasticity in transcription where noise in the expression of one gene is propagated to affect the noisiness of expression in a downstream gene. Noise propagation has been studied extensively where the authors examined sources of noise in a synthetic transcription cascade [2-5]. Such findings provide a solid understanding of noise in an artificially simple setting; however, there is clearly a need to develop experimental and data analysis techniques that permit the study of stochasticity in more complex regulatory systems, particular in endogenous gene networks.

Recently, the transcriptional regulatory network (TRN) of differentiating THP-1 cells has been characterized by integration of motif activity profiling, chromatin immunoprecipiation (ChIP) experiments, and by means of a RNAi perturbation matrix [6-8]. These studies demonstrated how the TRN is controlled by multiple regulators that exert their effects through the coordinated action of combinatorial transcription factors to elicit gene expression and cellular differentiation [9,10]. The architecture of the THP-1 regulatory network therefore allows cells to deal with the proper transmission of expression signals or take advantage of noise to modulate expression and thus function. However, these approaches revealed a snapshot of the THP-1 regulatory network while the endogenous gene networks are highly dynamic [1,11]. Therefore, expression profiling of single cells as they undergo cellular differentiation may provide clearer insights into the intricate dynamics of expression noise and its transmission to modulate gene expression.

During stem cell differentiation, the dynamic expression in space and time of key regulatory genes govern lineage specification of progenitor cells [11]. In this context, expression noise in transcription factors (TFs) might play an important role in regulating genes involved in development, stem cell maintenance and differentiation, and cell reprogramming [12-15]. The recent advancements in single-cell polymerase chain reaction (PCR) technology allow one to profile and analyze multiple genes in single cells and thereby provide the means to dissect cellular heterogeneity in various cellular systems. For instance, the multiplex single-cell expression analysis was used to measure cellular heterogeneity in rare populations isolated from different developmental stages [16-18], in cancer tissues [19,20], and cell reprogramming [15,21,22]. With these technical challenges surrounding multiplexed single-cell assays largely resolved, what remains to be explored is how gene expression noise is propagated and used within a defined network.

Therefore, this study describes the temporal dynamics of the THP-1 network in the context of single cells undergoing cellular differentiation. Key emphasis is given to stochastic effects of gene expression that can contribute to the regulation of cellular differentiation. Furthermore we establish modular structures of the THP-1 co-expression network and describe the dynamics of regulatory pathways in the context of RNAi-perturbations and transcription factor binding site predictions. The analysis identifies novel inter-dependent regulatory pathways between the transcriptional modules and further identifies a pivotal gene that modulates the maintenance and the differentiation of THP-1 cells.

## Results

### Single-cell profiling of THP-1 differentiation

**Figure 1:**
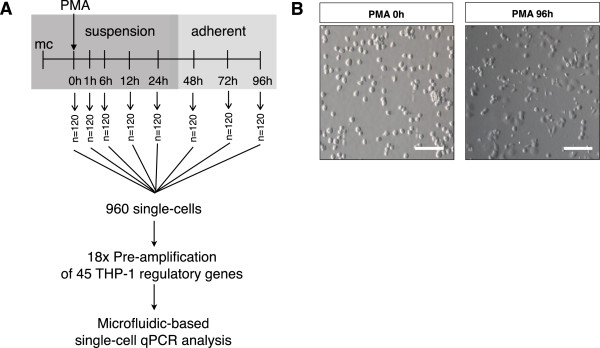
**THP-1 differentiation process. (A)** THP-1 cells are stimulated with PMA to induce monocyte/macrophage differentiation 1 day after medium change (mc). Single cells are manually picked at eight defined time points on three independent occasions (*n* = 120 per each time point). A total of 960 single cells were pre-amplified and profiled against 45 THP1 TFs. **(B)** Suspended THP-1 cells undergo differentiation upon PMA stimulation and a large majority (>95%) of cells adhere to the dish 96 h post induction with PMA. Scale bar = 50 μm.

Since our aim was to observe temporal variation of the THP-1 network during the differentiation process, we stimulated the THP-1 cells with phorbol 12-myristate 13-acetate (PMA) and manually picked 40 individual cells at eight distinct time points (0 h, 1 h, 6 h, 12 h, 24 h, 48 h, 72 h, and 96 h). This experiment was performed three independent times, resulting in a total of 120 single-cell expression profiles for each time point (Figure 1A). The endogenous control expressions in three experiments were validated and normalized for subsequent analyses (Additional file 1: Figures S1 and S2, Materials and Methods).

The human THP-1 myeloid monocytic leukemia cell line is an ideal model to study the temporal dynamics of single-cells because: (1) the cells in suspension undergo differentiation into a mature monocyte/macrophage-like phenotype upon simulation with PMA [6,23-25]; (2) the gene regulatory networks of differentiating THP-1 cells have been previously established (Additional file 1: Figure S3) [6,8]; and (3) the cells were sub-cloned for their ability to differentiate relatively homogeneously in response to PMA (Figure 1B) [6]. Thus applying micropipette technique together with microfluidic technologies we performed multiplexed PCR from single cells, allowing the simultaneous profiling 45 TFs represented in the THP-1 network (Additional file 2: Table S1).

### THP-1 cells are dynamically regulated during differentiation

**Figure 2:**
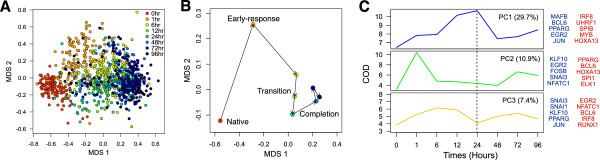
**Temporal dynamics of THP-1 differentiation. (A)** The multi-dimensional scaling (MDS) analysis reveals heterogeneous but population shifts in a time-dependent manner throughout the differentiation. **(B)** The centroids of each time point reveal temporal dynamics of cell populations throughout the time course, shifting from native, early-response, transition, to completion. **(C)** The coefficient of dispersion (COD) of three principal components (PC) and their variations shows distinct expression variation patterns throughout the time course and the associated TFs (blue TFs = positive contribution, red TFs = negative contribution).

In order to obtain a global perspective on noise in the cell population, single-cell expression profiles for all 45 TFs were reduced to a visualizable space using multiple dimension scaling (MDS) (Figure 2A). In inspecting the structure of MDS-reduced expression data, several important features that correlate with physiological characteristics of the cells can readily be discerned. The spatial progression throughout the differentiation, largely explained by the first axis, is a reflection of post-PMA stimulation in THP-1 cells. The MDS plot revealed wide scattering of individual cells that orderly shifted with time, suggesting that cellular heterogeneity is not random but tightly controlled. To further support this notion, the centroid projection of each time point revealed four distinct stages of the monocyte/macrophage differentiation: native, early-response, transition, and completion (Figure 2B). The early-response stage is clearly separated from the rest of the centroids, largely explained by the second MDS axis. Interestingly, the cell population coherently transits from 6 h to 24 h and suddenly shifts into a steady (that is, less variable) state, a period when the cells adhere to the dish and completes differentiation [6].

To further explore the control of cellular heterogeneity during monocyte/macrophage differentiation, we performed principal component analysis (PCA) and measured the coefficient of dispersion (COD) for the first three components at each time point (Figure 2C). The dispersion of the first component, which explained 29.7% of the cellular noise, steadily increased throughout the time course and peaked at 24 h post induction. This activation was then followed by rapid reduction and stability until 96 h post induction. Key pro-differentiation TFs such as PPARG and MAFB attributed mostly to the first component, while anti-differentiation factors such as IRF8 and MYB were the antithesis of this noise pattern. Moreover, the dispersion of the second component (10.9% variability explained) revealed a significant noise in the first hour while the third component (7.4%) revealed two transient dispersions separated at time point 24 h, a period when the cells adhere to the dish. This particular noise pattern substantiates the regulation of SNAIL superfamily of transcriptional repressors, SNAI1 and SNAI3, which regulate the changes in gene expression patterns that underlie components of the extracellular matrix, cell migration, and cell adhesion [26].

### Expression variability during THP-1 differentiation

**Figure 3:**
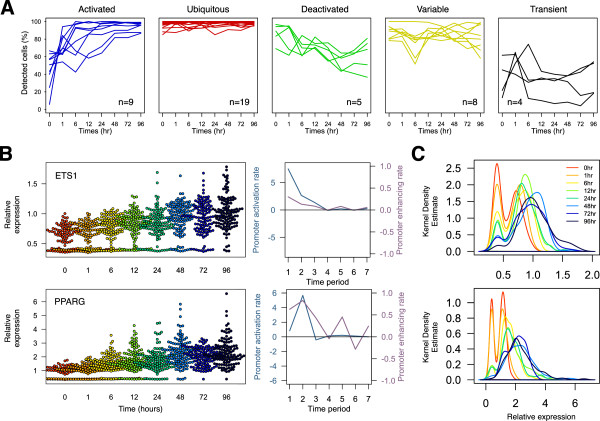
**Cell detection and expression dynamics throughout the time series. (A)** A systematic quantification of cells detected above threshold (relative expression >0.5) reveals five distinct clusters of cellular dynamics based on k-means clustering analysis (k = 5): activated, deactivated, transient, variable, and ubiquitous. **(B)** The ‘swarm-bee’ plots of ETS1 and PPARG reveal dynamic changes in cell number, transcript expression, and expression variation for each time point (rainbow). The changes in cell numbers and mean expression between every pairs of adjacent time points were calculated to explore differential modes of transcription: promoter-activation rate (blue line) and promoter-enhancement rate (purple line). **(C)** Density plots, based on Kernel Density Estimate (Gaussian) of ETS1 and PPARG, reveal the smooth distribution of single-cell expression across the time series, allowing population shift comparisons of the expression level across all time points.

In order to gain greater insights into the contribution towards cellular variation from individual TFs, we analyzed temporal changes in: (1) the number of cells expressing the transcripts; (2) differential TF expression; and (3) noise strength. We observed dramatic changes in the proportion of single cells expressing the transcript throughout the time course (Figure 3A). While 16 out of 45 TFs revealed ubiquitous transcript detection, 11 TFs exhibited ‘promoter activation’ where the sparsely expressed population either gradually or rapidly increased the percentage of positive cells throughout differentiation. Conversely, seven TFs showed reduction in the percentage of cells during differentiation.

While the proportions of detected cells were temporally changing, the mean expressions of detected cells were also changing, but at a different rate. To explore such differential modes of transcription, we analyzed the changes in cell numbers and their mean expressions between adjacent time points. Two distinct rates for transcript control were identified: (1) differential mean expression of detected cells across two time points, which describes the rate of promoter enhancement per hour; and (2) differential cell numbers across two time points, which describes the rate of promoter activation per hour, where the inducing signal enforced nothing-to-all response on the promoter. Based on this analysis, the PPARG-promoter was enhanced prior to the activation (Figure 3B). The changes in cell numbers between 0 h and 1 h was small in comparison to the difference in the mean expression in the same time period, which was >1.8-fold per h. This phenomenon suggests that PPARG was readily transcribed from already activated promoters upon PMA induction. However in the subsequent time frame (1 h and 6 h), we observed a significant increase in promoter activation (+5.8% per h) while the difference in expression only increased slightly when compared to the previous period. This suggests a prolonged delay in promoter activation possibly via epigenetic modification or essential expression of co-factors. In case of ETS1, the overall augmentation in gene expression was largely contributed by promoter activation from the onset of differentiation. These dynamic effects based on single-cell analysis indicate differential regulation of TF activation and TF enhancement on the promoter itself and explain the precise transcriptional regulation throughout the differentiation (Figure 3C).

### Temporal dynamics of noise during differentiation

**Figure 4:**
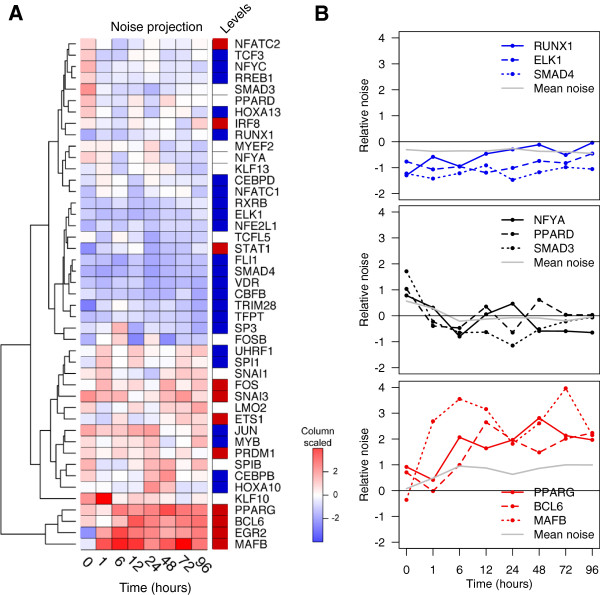
**Propagation of noise strength in respect to the THP-1 network. (A)** A heat map cluster representing scaled noise-strength for each TF across the time course (scaled z-score: red = noisy, blue = less noisy). Each TF is color-coded based on the topological layers of the THP-1 network (dark blue = upstream, white = midstream, dark red = downstream). **(B)** Noise projections of selected TFs and the mean noise strength for each topological layer (grey).

We then observed temporal dynamics in expression noise throughout the time series. At each time point, cell-to-cell variability was measured based on the expression variation across single cells divided by the mean expression. This relative noise of individual TFs as a function of time revealed strikingly temporal dynamics of transcription control (Figure 4A). It has been previously reported that expression noise can result from fluctuations of TFs upstream of the target gene in TRN [27-29]. Although synthetic circuits can be engineered to operate in isolation, gene circuits in nature are highly interconnected [1,11]. Based on the previously established THP-1 network [8], we calculated activating edge ratios (the number of downstream target genes divided by the number of upstream genes) in order to allocate the topological placement for each TF in the THP-1 network. The demarcations for each node were determined so that any TF with no upstream genes or edge ratio >4 were classified as ‘upstream’, and nodes with edge ratio <0.5, or has twice as many upstream genes as downstream genes, were classified as ‘downstream’. Any nodes falling in between these two ratios were classified as ‘midstream’ TFs (Additional file 1: Figure S3). Remarkably, downstream TFs revealed greater temporal noise throughout the differentiation whereas upstream TFs exhibited relatively low noise changes (*P* value <0.001). PPARG, MAFB, and BCL6, which belonged to the downstream layer, were among the top TFs with the increasing noise over time (Figure 4B). On the other hand, RUNX1, EKL1, and SMAD4, the upstream regulators of the THP-1 network, revealed relatively low variation throughout the differentiation. Interestingly, the midstream TFs revealed sporadic noise patterns where the average noise remained relatively still, possibly permitting the buffering of gene expression noise during the differentiation. This suggests that minor variations at the top of the network can propagate downwards to induce exponential variation to affect the TFs at the bottom of the network in the process of cellular differentiation.

### Dynamic rewiring of co-expression network during differentiation

**Figure 5:**
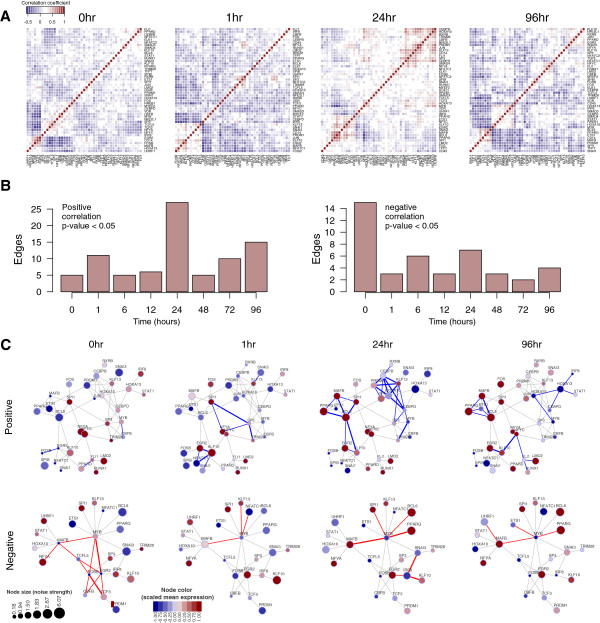
**Dynamic rewiring within the co-expression network during differentiation. (A)** Heatmap matrix of Spearman correlation analysis of 45 TFs across 120 single-cells at time points 0 h, 1 h, 24 h, and 96 h post differentiation and **(B)** quantification of significant edges (positive and negative) at each time point. **(C)** Co-expression networks showing putative relationship between TFs in all time points but highlighting activating or positive correlations (top row: blue edges) and antagonistic or negative correlations (bottom row: red edges) for time points 0 h, 1 h, 24 h, and 96 h post-PMA stimulation (*P* value <0.01). The node-colors indicate mean gene expression (scaled: blue = low expression; light brown = moderate expression; dark brown = high expression) while the node sizes indicate single-cell gene expression variations (small nodes = low variation; big nodes = high variation).

We then asked whether transcriptional relationships could be inferred from TFs with similar variation or noise patterns. At each time point, pairwise correlations were computed for 45 THP-1 TFs across 120 single cells from which we identified co-expressed modules of genes that were likely to be functionally co-regulated. Heat map representations of the topological overlap matrix for time point 0 h, 1 h, 24 h, and 96 h post-PMA induction demonstrate how these modules adopt different expression states during the time course (Figure 5A). The quantification of correlative edges revealed the highest number of interactions at time point 24 h post induction while the negatively correlated edges were greatest at time point 0 h (*P* value <0.05, Figure 5B). The differential numbers of regulatory edges indicate dynamic interplay of transcription factors to modulate differentiation, especially at time points 1 h and 24 h post PMA induction, whereas, the negative regulators of differentiation maintained the THP-1 steady state prior to differentiation (0 h). Moreover, the gene-network representations across the time series illustrate dynamic rewiring of transcription factors to establish time-specific network modules (Figure 5C, Additional file 1: Figure S4 for all time points).

### Exploration of gene co-expression network modules

**Figure 6:**
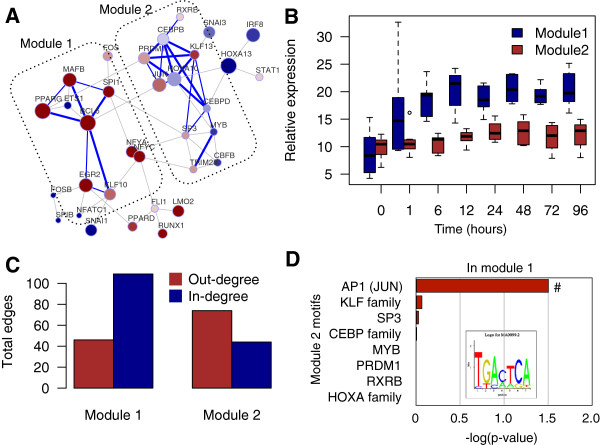
**Single-cell co-expression analysis identifies putative regulatory interactions between key modules. (A)** The co-expression network at 24 h reveals two modules that are highly interconnected. **(B)** The temporal changes in expression and variation of all TFs in module 1 and module 2. **(C)** The quantification of regulatory edges based on the RNAi-network reveals higher number of upstream genes for module 1 while opposite is true for module 2. **(D)** The transcription factor binding site (TFBS) analysis reveals a significant over-representation of AP1 (JUN) motif in module 1 as compared to all other motifs found in module 2 (# *P* value <0.05).

Expression noise is mediated by factors that bind at upstream promoter elements or influence the binding of other molecules to cis-regulatory elements within or near the promoter [11]. To describe the impact of expression noise at distinct levels of network organization and their temporal regulation, we analyzed: (1) differential expression analysis; (2) RNAi-perturbation matrix [7,8]; and (3) the transcription factor binding motif analysis [6]. Transcriptional modules are defined by a set of transcription factors that are strongly correlated with one another [30,31]. Based on this notion, the co-expression network analysis at 24 h revealed two transcriptional modules (Figure 6A). The averaged expression and variation of TFs depicted in each module revealed that module 2 remained steadily low while the variation increased time-dependently. Interestingly, the averaged expression of module 1 collectively increased >2-fold during the differentiation while the variation was vacillating throughout the time series (Figure 6B). It is a commonly held idea that negative-feedback provides a noise-reduction mechanism [1,11,32,33]. This seems to suggest that TFs in module 1 are likely to be involved in negative feedback which has a destabilizing effect, and result in dampened or enhanced expression patterns during the monocyte differentiation.

To better understand the inter-dependencies between the two network modules, we integrated the previously reported RNAi-based perturbation matrix [8]. The same 45 TFs described in this study were systematically knocked down in THP-1 cells and the differential expressions of the same 45 genes were quantified. The integration allowed us to enumerate the number of regulatory edges that were either upstream or downstream for each transcription factor (Additional file 1: Figure S3B). By directly incorporating this information, we revealed that the total number of *target* genes were significantly larger than the total number of *targeting* genes for module 1, whereas the opposite was true for module 2 (Figure 6C). This difference in the edge ratios further supports the notion that module 1 is regulated by external factors to illicit differentiation. The higher number of *target* genes in respect to *targeted* genes in module 2 suggests that TFs depicted in module 2 lie higher in the hierarchical structure of the monocyte transcriptional regulatory network and may act as a master modulator of monocyte/macrophage maintenance [11].

The level of noise might also be directly impacted by other factors such as the sequences of the promoter itself. To further exploit the regulatory promoter elements of the modules, we analyzed the over-representation of predicted transcription factor binding sites occurring in the proximal promoter regions of module 1 factors. Considering all potential binding sites for module 2, the motif analysis revealed a significant enrichment of AP1 (JUN) binding sites in the proximal promoter regions of module 1 (*P* value = 0.017; Figure 6D). Indeed almost all but KLF10 in module 1 exhibited AP1 binding site in the proximal promoter. No significant motifs were found when the analysis was reversed to predict binding sites in module 2.

### Functional characterization of MYB as a network hinge

**Figure 7:**
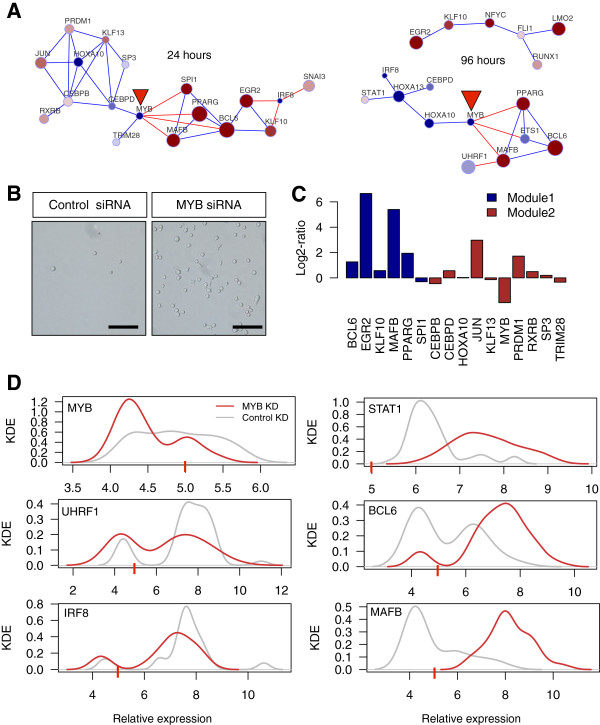
**The integration of co-expression networks reveals MYB as a pivotal driver of monocyte differentiation. (A)** Pairing of positive and negative co-expression networks at time points 24 h and also at 96 h reveals MYB as a pivotal driver (red arrow), hingeing two antagonistic network modules. **(B)** siRNA mediated knockdown of MYB in THP-1 cells leads to cellular differentiation observed by cell adherence to the dish after several washing steps. **(C)** Depletion of MYB in THP-1 cells induces expression of key pro-differentiation factors such as EGR2, MAFB, and PPARG as compared to siRNA control. **(D)** MYB knockdown followed by single-cell gene expression analysis reveals various modes of gene expression regulation and distribution (grey line = control (*n* = 43), brown line = MYB siRNA knockdown (*n* = 43), red bar = detection threshold at 5). Scale bar = 50 μm.

Stochastiticity in gene expression was originally hypothesized to be harmful to cells as it represents an irregularity or lack of stability of gene expression levels [1]. More recently, however, there has been speculation that stochasticity has a constructive role in development and cellular differentiation in higher organisms [34,35]. To uncover the modular basis for stochasticity during THP-1 differentiation, we aggregated both the positive and negative networks at 24 h and also at 96 h post induction (Figure 7A). Prominently at 24 h, the same two modules observed in Figure 6 were separated by negatively correlated edges anchored by a key inhibitor of cellular differentiation, MYB. MYB is a proto-oncogene protein which consists of a central transcriptional activation domain and a C-terminal domain involved in transcriptional repression [36]. The combined co-expression network demonstrates both active and suppressive role of MYB and suggests the gene as a regulatory hinge that is essential to both monocyte maintenance and differentiation. To confirm this, we suppressed the MYB transcript in THP-1 cells in the absence of PMA. Notably within the first 48 h, siRNA transfected THP-1 cells adhered to the dish, indicative of monocyte/macrophage differentiation (Figure 7B). Moreover, the conventional qRT-PCR expression analysis using bulk mRNA of MYB suppressed THP-1 cells revealed increased expression of pro-differentiation genes such as MAFB, EGR2, and PPARG as compared to control siRNA (Figure 7C).

To further elucidate the transcriptional control of MYB, we performed MYB siRNA transfection followed by single-cell gene expression analysis. As expected, MYB expression was downregulated, but interestingly, the broad MYB expression in the control group was lost in the MYB siRNA transfected group (that is, high noise to less noise) (Figure 7D). In contrast, STAT1 shifted from low noise strength to high noise strength upon MYB suppression, revealing dynamic regulation of MYB, not only in the gene expression but also in the expression heterogeneity required for cellular differentiation. Additional transcription factors such as BCL6 revealed a shift in expression modality (from bi-modal to uni-modal), while MAFB revealed a population shift in expression distribution (uni-modal to uni-modal) upon MYB suppression. Taken together, oligo transfections followed by single-cell gene expression profiling revealed, for the first time, a highly dynamic regulation of transcription expression and noise patterns, which would not have been possible with bulk culture mRNA expression profiling.

## Discussion

Here we applied single-cell expression analysis to investigate the population heterogeneity of THP-1 cells undergoing differentiation. We characterized 45 previously defined TFs associated with the THP-1 network in terms of their cooperative relationships formed after application of a differentiating agent. We showed that single-cell expressions are noisy but are tightly controlled during the differentiation, leading to both ‘noisy’ and ‘stable’ phases based on the single-cell dispersion analysis. Moreover, we revealed time specific co-expression modules that are dynamic and cooperatively regulated by nearby modules and confirmed MYB as a key modulator of THP1 maintenance and differentiation.

Coordinated and complex transcriptional responses generally involve multiple regulatory interactions organized into gene circuits [1,6,11]. In such an organization, the information sensed by upstream layer of circuits is relayed to activate downstream layer [37]. Here, we revealed that not only do these circuits modulate expression but also noise may be transmitted through the network where the midstream components buffer the noise to elicit gene-specific activation in the network. Interestingly, we revealed that suppression of MYB transcript in THP-1 not only led to cellular differentiation but also induced a substantial increase in expression heterogeneity of STAT1, a downstream transcription factor. Essentially, the interconnected gene circuits, or network modules, seems to influence the sensitivity to noise. Furthermore, a DNA motif with affinity for the TATA-box binding protein (TBP) has been shown to contribute significantly to high noise [38]. In this respect, the DNA motif with affinity for AP1 was significantly enriched in network modules demonstrating the temporal variations are not contributed individually but via modules in a circuit.

Slow transitions between promoter states are known to be particularly important in eukaryotic gene expression, since the presence of nucleosomes that cause DNA to be packed into units of chromatin generally make promoters inaccessible to the transcriptional complex [39]. Mixed populations and bimodal population distributions, which were prevalent for most pro-differentiation TFs at time point 0 h, might arise from slow promoter transition rates. Interestingly, the differential promoter activation rates revealed insights into transcript regulation where the PPARG promoter was slow to transit into active states possibly due to epigenetic regulation [40]. Moreover, the temporal dynamics of single-cell expressions demonstrate that PMA stimulation changes expression in which all cells respond in proportion to the inducing signal or changes the probability of a stochastic all-or-nothing response in an individual cell.

The intrinsic stochasticity that regulates the biochemical process of producing a single transcript is not the only source of variability in gene expression. Factors such as gene regulatory signals, the abundance of polymerases and ribosomes, and the strength of their binding, availability of TFs, and cell cycle can inevitably change cell size and activity [41,42]. In the present study, extrinsic noise from transcript abundance was normalized by means of GAPDH expression (Additional file 1: Figure S2) where ubiquitous myeloid-lineage specific TFs such as SPI1 and GAPDH were highly correlated. Moreover, it is understood that before initiating cell differentiation, PMA treatment first induce inhibition of cell growth at G1-phase of the cell cycle [43]. Therefore, the increased fluctuations in gene expression during differentiation provide a mechanism for distinct physiological states, and could therefore increase the precision that is necessary for single-cell analysis.

## Conclusion

Taken together, the insights we have obtained in this study are a consequence of analyzing a defined set of transcription factors in parallel at the single-cell level over the time series. It will also be of great interest to integrate the information from correlated expressions with other types of networks (for example, protein-protein interactions). Our high throughput single-cell analysis offers intriguing new insights into the mammalian gene regulatory interaction, and the application of these methods to other biological systems will further clarify the underlying molecular mechanism controlling cellular maintenance and differentiation.

## Materials and methods

### Cell culture and PMA stimulation

THP-1 cell line was sub-cloned by limiting dilution and one clone was selected for ability to differentiated relatively homogenously in response to PMA [6]. THP-1 cells were cultured in RPMI1640 (Invitrogen), 10% FBS, penicillin/streptomycin (Invitrogen), 10 mM HEPES, 1 mM Sodium Pyruvate and 50 μM 2-mercaptoethanol at 37°C, and 5% CO_2_. One day prior to PMA stimulation, fresh cultured medium was replaced. THP-1 cells (2 × 10^5^) were treated with 30 ng/mL PMA (Sigma) over a time course of 96 h. To demonstrate that cells are differentiated into monocytes, flow cytometric analysis with anti-human CD14, CD45, and CD11B antibodies (all Biolegend) were performed at eight time points post PMA induction (Additional file 1: Figure S5).

### Single-cell isolation and pre-amplification

Each single cell was isolated by hand-pipetting using micro-dispenser (Drummond) under the phase contrast microscope. Cells after 48 h PMA stimulation were treated with 0.05% Trypsin/EDTA (Wako) for 20 min at 37°C to detach the cells from the dish. All cells were washed with PBS to remove any trace of medium or trypsin/EDTA. Individually picked single cells were subjected to pre-amplification mixture containing 0.2 mM dNTP, 1.6 mM MgSO_4_, 0.5 μM gene-specific primers, RNase Free Water (Ambion), 0.6 U RNaseOUT (Invitrogen), and SuperScript III RT/Platinum TaqMix. Pre-amplification was performed at 55°C for 25 min and 95°C for 2 min followed by 18 cycles of 95°C for 15 s and 60°C for 4 min. After pre-amplification, cDNA was diluted 1:4 with RNase Free Water and stored in −80°C until needed.

### Real-Time PCR

Real-Time PCR was carried out by the BioMark™ Dynamic Arrays of Fluidigm. 1× FastStart Universal Probe Master (ROX), 1× GE Sample Loading Reagent, and each of diluted pre-amplified cDNA was prepared. And 5 μL assay mix containing 2.5 μM primer mix, 1.3 μM UPL probe, 1× Assay Loading Reagent, and RNase Free Water was prepared. An IFC controller was used to prime the fluidics array with control line and then with samples and assay mixes. After loading, the array was placed in the BioMark Instrument for PCR at 95°C for 10 min, followed by 40 cycles at 95°C for 15 s, 70°C for 5 s, and 60°C for 1 min.

### Single-cell gene expression analysis

Amplification curves derived from the Fluidigm Data Collection Software were filtered using a threshold of 0.65 and Ct threshold mode was set to global. From the initial 1,041 single cells profiled, single cells expressing >2 standard deviations from mean GAPDH expression and mean SPI1 expression were deemed unviable and thus omitted from further downstream analyses. Thereafter, 120 single cells from each time point were randomly selected and fixed for the analyses (120 single cells × 8 time points = 960 single cells). Expression Ct values >36 were set to 40. Then, the relative gene expression was calculated by subtracting Ct_gene_ by Ct_GADPH_ followed by inverse to make the data more intuitive. The inverse-normalized value of 36 is 0.5 (or 5), a cutoff used to determined transcript-positive and -negative single cells (Additional file 1: Figure S1). All of the normalized Ct values are available in Additional file 3.

Multiple dimension scaling (MDS; Pearson correlated) and principal component analysis (PCA) were performed in R [44]. COD was calculated by median absolute variation divided by median. Hierarchical clustering and co-expression matrix between pairs of TFs were tested with Spearman rank correlation (false discovery rate <0.05). Gene regulatory circuits were illustrated using the RedeR [45] package in R Bioconductor.

For DNA motif analysis, we downloaded the transcription factor binding site predictions previously calculated as part of the FANTOM4 project [6] using motifs stored in the SwissRegulon database [46,47]. For each promoter, we summed the posterior probabilities of the binding sites predicted for each motif to obtain, for each promoter, its *a posteriori* number of binding sites of the given motif. Assuming that for a given number of motif across promoters follows a Poisson distribution, we calculated the tail probability of the *a posteriori* number of binding sites for the promoters located at the 5′ end of the genes of interest. In practice, we found that the variance of the distribution was slightly lower than its mean, implying that the true distribution is under-dispersed compared to the Poisson distribution, and hence the *P* values we calculated are conservative.

### siRNA transfection and RT-PCR analysis

Reverse transfection of 1 × 10^6^ THP-1 cells in each 60-mm cell culture dish was performed with 20 nM of stealth negative control RNA, MYB siRNA (GCCGCAGCCAUUCAGAGACACUAUA) in Opti-MEM and Lipofectamine 2000 (Invitrogen), according to the manufacturer’s instructions. Forty-eight hours post transfection, either single cells or total RNA from bulk culture were extracted. Oligo-transfected single cells were profiled using the aforementioned methods. As regards to bulk samples, total RNA was isolated using miRNAeasy kit (Qiagen) according to manufacturer’s instructions. Reverse transcription of total RNA was achieved with PrimeScript™ reserve transcriptase (Takara) and random hexamers in according with the manufacturer’s protocol GAPDH mRNA was used as a control for data normalization. PCR amplification was performed on ABI PRISM® 7500 (Applied Biosystems). For amplification, SYBR Premix EX Taq™ II (Takara) was used as instructed in the manual. Changes of gene expression were determined using the 2^-ΔΔCt^ method [48].

## Abbreviations

ChIP: Chromatin immunoprecipitation; COD: Coefficient of dispersion; DNA: Deoxyribonucleic acid; FANTOM: The functional annotation of the mammalian genome; KDE: Kernel density estimate; MDS: Multiple dimensional scaling; PCA: Principal component analysis; PCR: Polymerase chain reaction; PMA: Phorbol 12-myristate 13-acetate; qRT-PCR: Quantitative reverse transcription polymerase chain reaction; RNA: Ribonucleic acid; RNAi: Ribonucleic acid interference; siRNA: Small interfering ribonucleic acid; TBP: TATA-box binding protein; TF: Transcription factor; TFBS: Transcription factor binding site; THP-1: Human monocytic cell line from an acute monocytic leukemia patient; TRN: Transcriptional regulatory network.

## Competing interests

The authors declare no competing financial interests.

## Authors’ contributions

JWS, MK, and TK designed and performed single-cell experiments. JWS, MdH, and JCM performed bioinformatics analysis. JWS, TK, JCM, and MdH wrote the paper. YT and MK performed perturbation experiments. HS, PC, YH, and JWS conceived the study and oversaw the project. All authors read and approved the final manuscript.

## Supplementary Material

Additional file 1 Supplementary information of Figures S1-S5. Click here for file

Additional file 2: Table S1 List of THP1 network genes and primers sequences with associated UPL probes for specificity. Click here for file

Additional file 3 Normalized single-cell gene expression dataset (120 single cells × 8 time points). Click here for file
