# Supplementary material for: Temporal dynamics and transcriptional control using single-cell gene expression analysis
Source: Genome Biol. 2013 Oct 24;14(10):R118. doi: 10.1186/gb-2013-14-10-r118 (PMC4015031; doi:10.1186/gb-2013-14-10-r118)
Supplement: Additional file 1 — Supplementary information of Figures S1-S5. [file gb-2013-14-10-r118-S1.pdf]

Supplementary Information

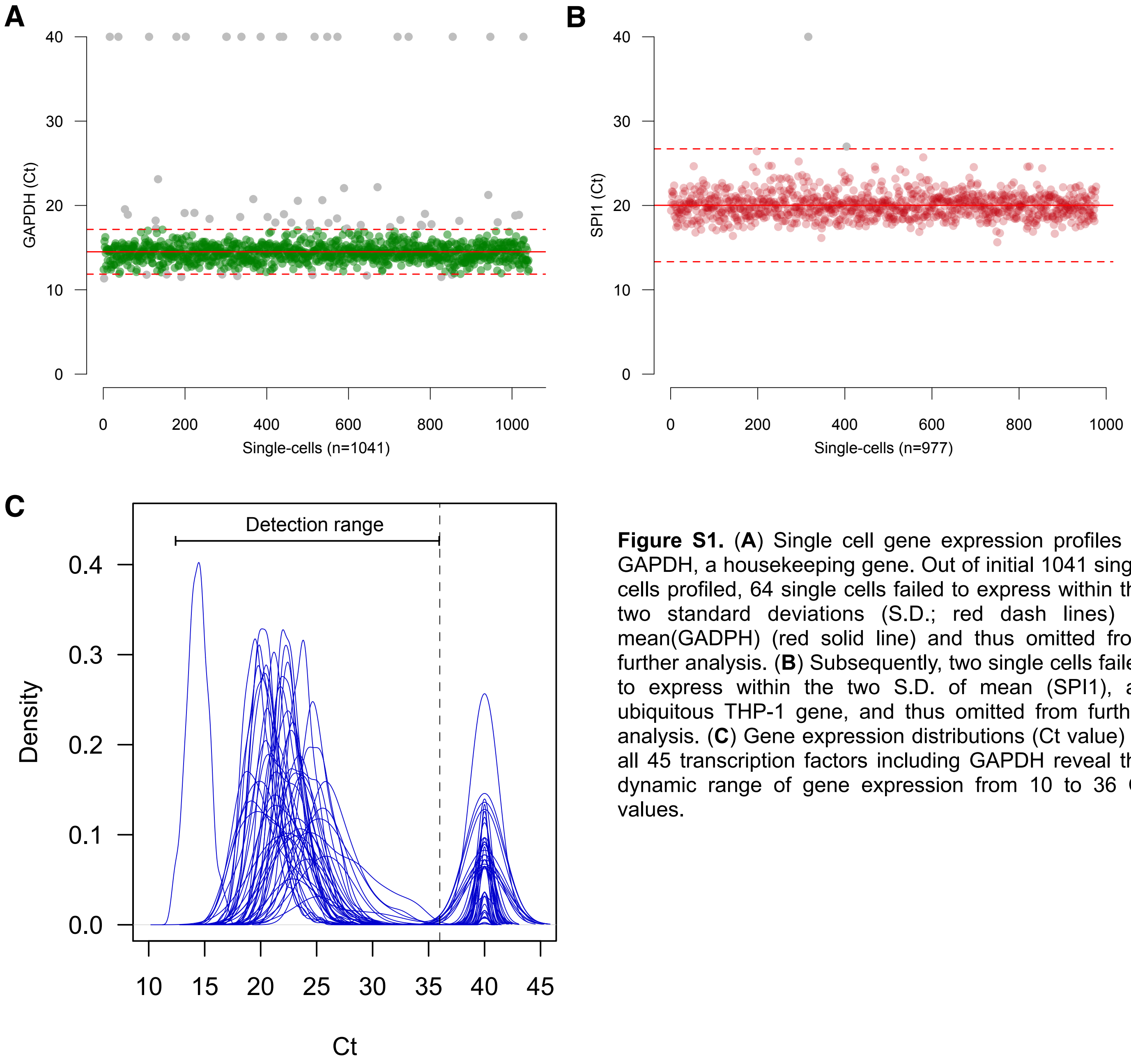

Figure S1. (**A**) Single cell gene expression profiles of GAPDH, a housekeeping gene. Out of initial 1041 single cells profiled, 64 single cells failed to express within the two standard deviations (S.D.; red dash lines) of mean(GADPH) (red solid line) and thus omitted from further analysis. (**B**) Subsequently, two single cells failed to express within the two S.D. of mean (SPI1), an ubiquitous THP-1 gene, and thus omitted from further analysis. (**C**) Gene expression distributions (Ct value) of all 45 transcription factors including GAPDH reveal the dynamic range of gene expression from 10 to 36 Ct values.

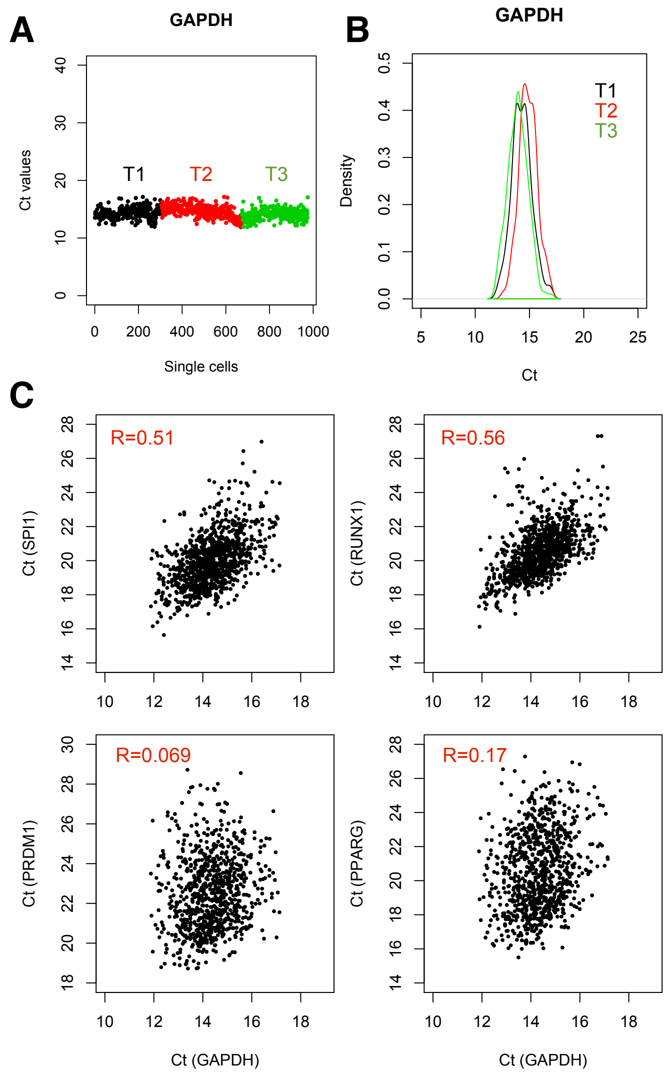

D

Normalization of single-cell PCR based on GAPDH expression

For each gene, Ct values of GAPDH is subtracted and inversed.

$$\text{Ct-norm} = \frac{1}{\text{Ct(gene)} - \text{Ct(GAPDH)}}$$

Figure S2. Control expression across three independent trials and normalization. (**A, B**) GAPDH was used as endogenous control. In all three independent trials (T1, T2, T3), GAPDH Ct values ranged from 11 - 17. (**C**) When GAPDH expression was compared to myeloid-lineage specific transcription factors, SPI1 and RUNX1, we observed greater correlation values when compared to non-myeloid-lineage genes: PRDM1 and PPARG. This suggested that the GADPH expression correlated with the overall quantity of transcripts per cell. (**D**) Therefore, gene expressions were normalized by taking the inverse of differential expression to GAPDH.

A

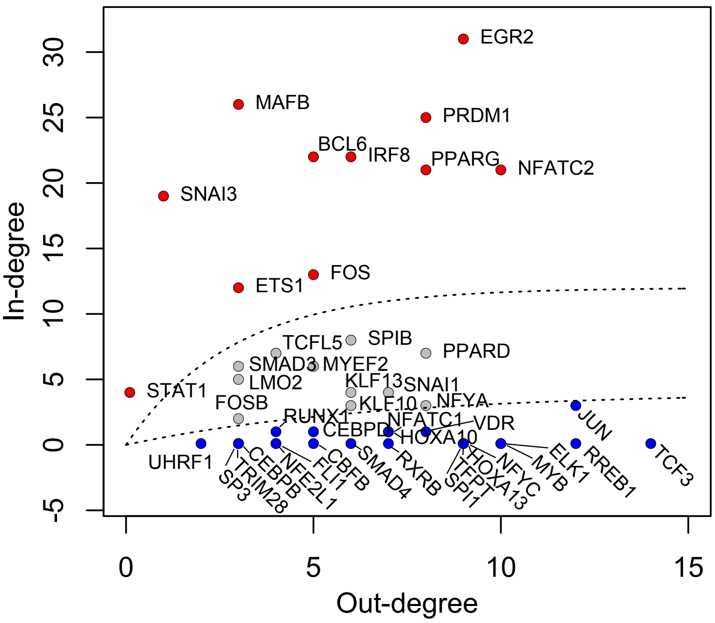

B

Upstream	Out-degree	In-degree
CBFB	5	0
CEBPB	3	0
CEBPD	5	1
ELK1	10	0
FLI1	4	0
HOXA10	7	1
HOXA13	9	0
JUN	12	3
MYB	10	0
NFATC1	7	1
NFE2L1	4	0
NFYC	9	0
RREB1	12	0
RUNX1	4	1
RXRB	7	0
SMAD4	6	0
SP3	3	0
SPI1	9	0
TCF3	14	0
TFPT	9	0
TRIM28	3	0
UHRF1	2	0
VDR	8	1

Downstream	Out-degree	In-degree
BCL6	5	22
EGR2	9	31
ETS1	3	12
FOS	5	13
IRF8	6	22
MAFB	3	26
NFATC2	10	21
PPARG	8	21
PRDM1	8	25
SNAI3	1	19
STAT1	0	4

Midstream	Out-degree	In-degree
FOSB	3	2
KLF10	6	3
KLF13	6	4
LMO2	3	5
MYEF2	5	6
NFYA	8	3
PPARD	8	7
SMAD3	3	6
SNAI1	7	4
SPIB	6	8
TCFL5	4	7

Figure S3. THP-1 transcriptional regulatory network (TRN) based on RNAi perturbation matrix. (**A**) Tomaru Y. et al. 2009 performed a systematic knockdown of all 45 transcription factors (TFs) investigated in this study in THP-1 cell lines. Individual 45 knockdowns followed by gene expression profiling of the same 45 genes led to illustrate a THP-1 Transcriptional Regulatory Network based on RNAi perturbation matrix (differential expression of two-fold or more, p-value < 0.05). The number of in-degree and out-degree edges per TF led to the classification of upstream, midstream, and downstream genes in respect to the TRN. (B) A table representing the number of in-degree and out-degree edges per transcription factor (blue: upstream, grey: midstream, red: downstream).

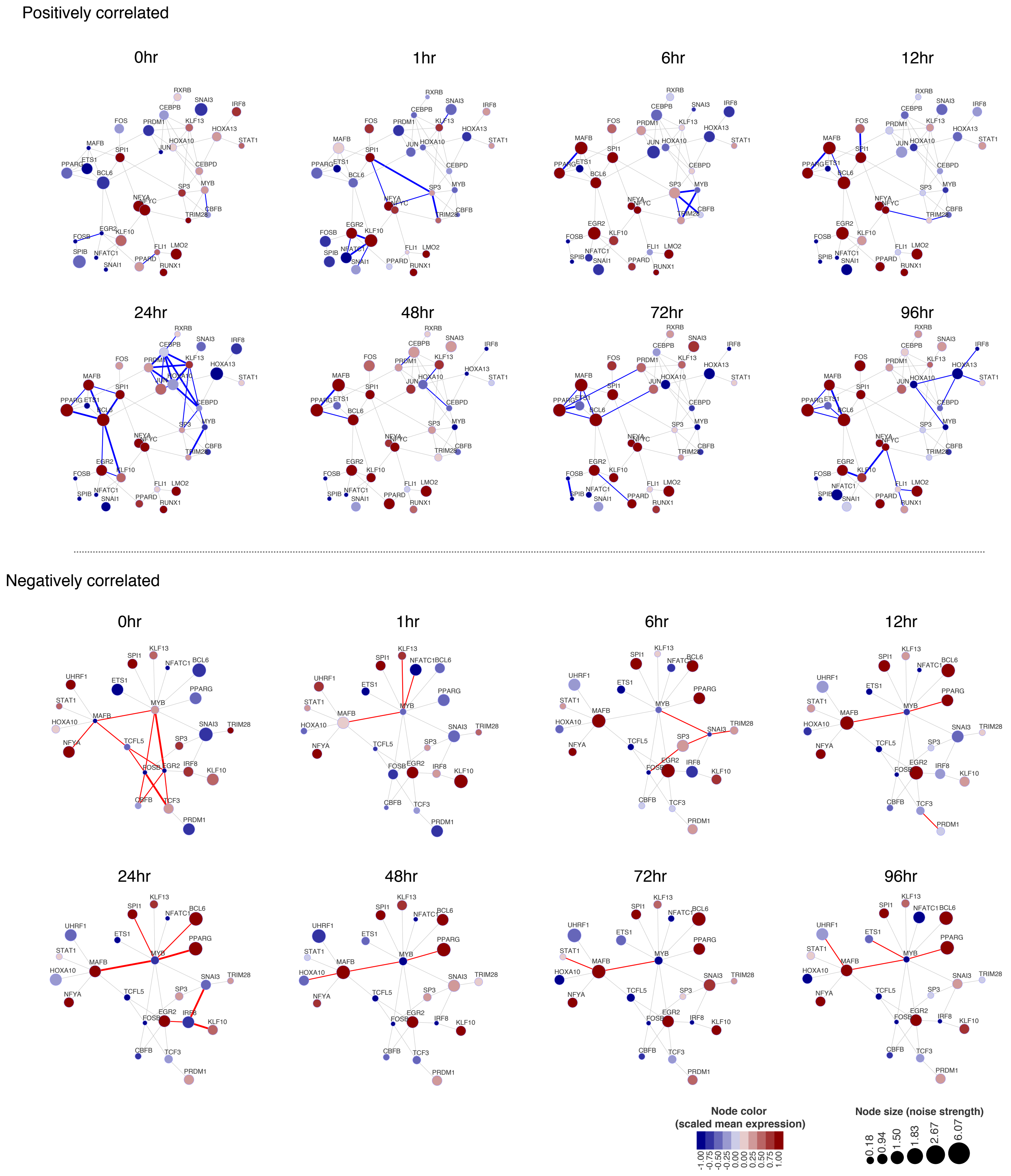

Figure S4. Co-expression networks showing putative relationship between TFs in all time points but highlighting or positive correlations (top row: blue edges) and antagonistic or negative correlations (bottom row: red edges) for all time points post PMA stimulation. The node-colors indicate changes in gene expression (scaled: blue=low expression; light brown= moderate expression; dark brown=high expression) while the node-sizes indicate variation (small nodes=low variation; big nodes=high variation).

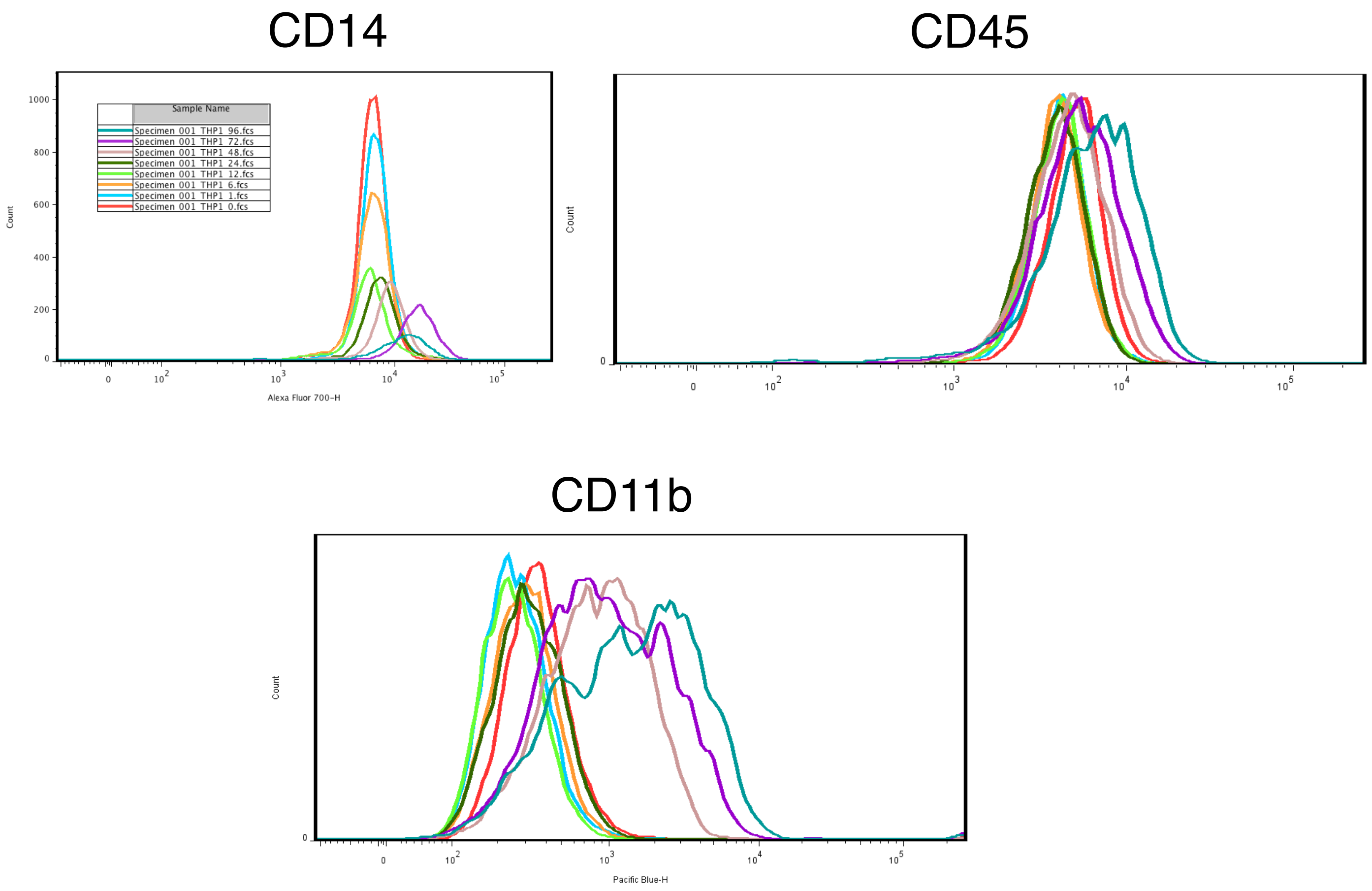

Figure S5. Flow cytometry analysis during THP-1 differentiation of CD14, CD45 and CD11b. Induction of PMA leads to increase in CD14 protein expression while CD45 expression stays relatively similar. Moreover, CD11b protein expression is readily expressed from time point 48 hrs post PMA induction.
