# Supplementary material for: Temporal dynamics and transcriptional control using single-cell gene expression analysis
Source: Genome Biol. 2013 Oct 24;14(10):R118. doi: 10.1186/gb-2013-14-10-r118 (PMC4015031; doi:10.1186/gb-2013-14-10-r118)
Supplement: Additional file 2: Table S1 — List of THP1 network genes and primers sequences with associated UPL probes for specificity. [file gb-2013-14-10-r118-S2.pdf]

Supplementary table 1. List of THP1 network genes and primers sequences with associated UPL probes for specificity

	Gene	Direction	Sequence	UPL probe No.
**1**	KLF10	Sense	agccaaccatgctcaacttc	#67
		Anti-sense	ctcttttggcctttcagaaatc	#67
**2**	KLF13	Sense	gtgcccgcttcttaccact	#33
		Anti-sense	gaagtcaccaccacgctgt	#33
**3**	LMO2	Sense	ttcaacctgaacttgcagtagc	#34
		Anti-sense	gcttccctctgtctctggttt	#34
**4**	BCL6	Sense	ctgcagacccacagtgaca	#12
		Anti-sense	tgcaacgatagggtttctca	#12
**5**	CBFB	Sense	ttcacagctgctggcagtaa	#9
		Anti-sense	gctgtgctattaattaacgaagtttg	#9
**6**	CEBPB	Sense	ctggagacgcagcacaag	#1
		Anti-sense	acagctgctccaccttcttc	#1
**7**	CEBPD	Sense	ggacataggagcgcaaagaa	#64
		Anti-sense	gcttctctcgcagtttagtgg	#64
**8**	EGR2	Sense	ttgaccagatgaacggagtg	#3
		Anti-sense	tggtttctaggtgcagagacg	#3
**9**	ELK1	Sense	tgcttcctacgcatacattga	#16
		Anti-sense	ggtgctccagaagtgaatgc	#16
**10**	ETS1	Sense	acaagcctgtcattcctgct	#86
		Anti-sense	gtaattccagaagaaactgccatag	#86
**11**	FLI1	Sense	aacgatcagtaagaatacagagcaac	#20
		Anti-sense	gctaggcgactgctggtc	#20
**12**	FOS	Sense	ctaccactcacccgcagact	#67
		Anti-sense	aggtccgtgcagaagtcct	#67
**13**	FOSB	Sense	gagctgaccgaccgactc	#69
		Anti-sense	ccgactccagctctgcttt	#69
**14**	HOXA10	Sense	gttttgcacaagaaatgtcagc	#32
		Anti-sense	gacattgttgtgggataatttgg	#32
**15**	HOXA13	Sense	atgtactgccccaaagagca	#75
		Anti-sense	gcttctttctccccctcctat	#75
**16**	IRF8	Sense	gaggtggtccaggtcttcg	#20
		Anti-sense	cggccctggctgttatag	#20
**17**	JUN	Sense	ccaaaggatagtgcgatgttt	#19
		Anti-sense	ctgtccctctccactgcaac	#19
**18**	MAFB	Sense	agggaagctgccaagctc	#82
		Anti-sense	atttgaccataagacaaggctgt	#82
**19**	MYB	Sense	cccttactgaagaaaatcaaacaag	#48
		Anti-sense	tgcgtgaacagttgggtatt	#48
**20**	MYEF2	Sense	tcttctaacatgggtccagtagg	#31
		Anti-sense	cccattcctccagtcacact	#31
**21**	NFATC1	Sense	atgaagtcagcggaggaaga	#88
		Anti-sense	gtgtggaggtctgaaggttgt	#88
**22**	NFATC2	Sense	tattacctgcgggggtgac	#68
		Anti-sense	ccagctaaggtgtgtgtctatca	#68
**23**	NFE2L1	Sense	gacgccgagctaagcagtt	#25
		Anti-sense	cgtcttgggtcagagtgtcc	#25
**24**	NFYA	Sense	ggcaagcccgagctaaac	#29
		Anti-sense	ccgagactcatgcaggtattt	#29

	Gene	Direction	Sequence	UPL probe No.
**25**	NFYC	Sense	cgaaggtgcctgaaagaaac	#56
		Anti-sense	tccatctgtgaactggctga	#56
**26**	PPARD	Sense	cggtgttcatgcatgtgag	#78
		Anti-sense	agctgcgctcacacttctc	#78
**27**	PPARG	Sense	gacaggaaagacaacagacaaatc	#7
		Anti-sense	ggggtgatgtgtttgaacttg	#7
**28**	PRDM1	Sense	ccgaccaaagcctcaaga	#66
		Anti-sense	ccacaggggacaccgtatt	#66
**29**	RREB1	Sense	aagatcacctgtccccactg	#24
		Anti-sense	caagggaagggtttctgacc	#24
**30**	RUNX1	Sense	acaaacccaccgcaagtc	#21
		Anti-sense	catctagtttctgccgatgtctt	#21
**31**	RXRB	Sense	agctcccccaggattctc	#66
		Anti-sense	ccagggagtgacactgttgag	#66
**32**	SMAD3	Sense	caccacgcagaacgtcaa	#9
		Anti-sense	gatgggacacctgcaacc	#9
**33**	SMAD4	Sense	cctgtggcttccacaagtc	#46
		Anti-sense	ctgatgctatctgcaacagtcc	#46
**34**	SNAI1	Sense	gctgcaggactctaatccaga	#11
		Anti-sense	atctccggaggtgggatg	#11
**35**	SNAI3	Sense	ccacagggtccccaactac	#28
		Anti-sense	gagcaggcaccattgatttc	#28
**36**	SP3	Sense	ttgcacctgtcccaactgta	#31
		Anti-sense	tgttgcttctttttcccaaga	#31
**37**	SPI1	Sense	caggggatctgaccgactc	#42
		Anti-sense	gcaccaggtcttctgatgg	#42
**38**	SPIB	Sense	gcataccccacggagaact	#23
		Anti-sense	cggtaagtcttcctcctctgatag	#23
**39**	STAT1	Sense	tgagttgatttctgtgtctgaagtt	#32
		Anti-sense	acacctcgtcaaactcctcag	#32
**40**	TCF3	Sense	gagaagccccagaccaaact	#64
		Anti-sense	ggattcaggttccgctctc	#64
**41**	TCFL5	Sense	gagactgacaaggccacaact	#86
		Anti-sense	ccgcaaaatacgctctcaa	#86
**42**	TFPT	Sense	tccatcaggtgcagaggata	#18
		Anti-sense	cgtaggagtccagcactctcat	#18
**43**	TRIM28	Sense	tggtcaatgatgcccaga	#1
		Anti-sense	cttggtcatggtccagtgc	#1
**44**	UHRF1	Sense	aagatccaggagctgttcca	#36
		Anti-sense	aagagggtatggccgtcct	#36
**45**	VDR	Sense	acatcggcatgatgaagga	#6
		Anti-sense	ttccgcttcaggatcatctc	#6
**46**	GAPDH	Sense	agccacatcgctcagacac	#60
		Anti-sense	gcccaatacgaccaaatcc	#60
